# Comparison of the WIDAR application with a manual photogrammetry method of creating 3D models of cadaveric hearts

**DOI:** 10.1007/s40670-024-02131-8

**Published:** 2024-08-02

**Authors:** Nathan A. Tullos

**Affiliations:** Department of Advanced Biomedical Education, University of Mississippi Medical Center, Jackson, MS USA

**Keywords:** 3D models, Medical students, Undergraduate medical education, Anatomy

## Abstract

Novel methods of bringing gross anatomy laboratory experiences into the lecture setting may offset decreased contact hours. The iOS and Android photogrammetry application WIDAR is a time and cost-effective method for generating 3D digital models of donor specimens for use in medical education.

Photogrammetry is an effective method of generating accurate 3D digital models of anatomic structures [1, 2]. Research demonstrates that the use of 3D models can improve student performance compared to 2D illustrations [3, 4]. Digital models can be used in a variety of settings, classroom lectures, problem-based small groups, as supplements to dissection, or as components of dissection free course design.

Decreasing student contact hours continue to drive development of novel methods for bringing laboratory experiences into the classroom. Additionally, cutting student dissection time may affect the quality of the dissection experience and could lead to worse student outcomes [5, 6]. While students prefer active learning to traditional lecture [7], systems-based integrated curricula make it difficult to schedule laboratory experiences throughout the preclinical years. Therefore, the capability to generate accurate 3D digital models of cadaveric structures can bridge the gap between lecture and laboratory.

A cloud-based photogrammetry application was compared to a previously described manual method of photogrammetry [8]. Three student-dissected donor hearts were used for this study. For the manual method, two hundred photographs from prescribed angles were taken using the Manual Camera DSLR Pro application (https://play.google.com) and 3D models were assembled using the Agisoft Metashape photogrammetry software (www.agisoft.com). Textures were embedded onto the manual model using the Blender software (www.blender.org). This method was compared to the WIDAR smartphone application (www.widar.com). Two hundred photographs were taken within the application and uploaded to the WIDAR server for model generation with textures embedded. For both methods, the photographs were taken using a Samsung Galaxy S21 smartphone.

The mean model production time of the manual method was 2 h and 39 min (02:39:03) while the total WIDAR time was 27 min (0:27:35). The reduction was due to decreased generation time rather than photography time (photography time Manual 0:19:10 vs WIDAR 0:13:51; generation time: Manual 02:19:51 vs WIDAR 0:13:44). Additionally, the mean file size of the WIDAR model was smaller than the manual model (Manual 23.5 MB vs WIDAR 9.5 MB). The reduction in time and file size in the WIDAR model did not decrease the aesthetic quality or resolution compared to the manual model (Fig. 1). The WIDAR method greatly reduces the barrier to photogrammetry in medical education. The technique is easy to learn, does not require graphics-intensive computing, or specialized skills. Increased implementation of active learning using innovative modeling techniques may lead to improved student performance.

**Fig. 1:**
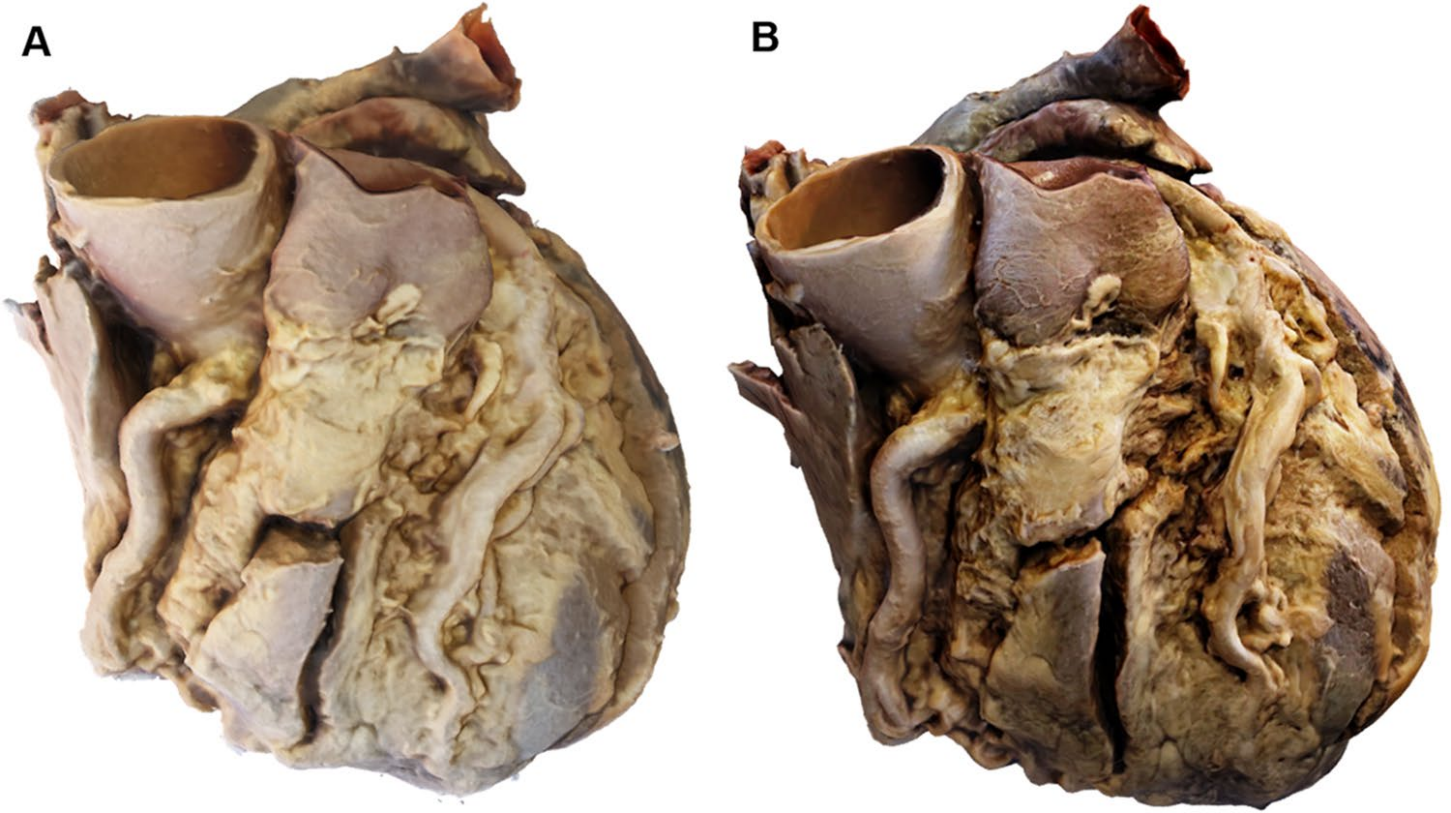
Photogrammetry-produced 3D digital models of a student-dissected heart using **A** the manual method and **B** the WIDAR method

There are limitations of this study to consider. This project was a descriptive study examining the practical limitations of model generation and did not investigate the effect of WIDAR models on student engagement or performance. Secondly, the manual method provides full control of the model generation parameters and data. While 3D digital models, like photographs, fall under the anatomical use outlined in our institution’s authorization for body donation, sensitive or identifying structures such as the pelvis or face should be restricted to manual photogrammetry. Despite these limitations, easy-to-use methods of model generation such as WIDAR can lead to increased implementation of 3D digital models in lecture, problem-based learning, and remediation sessions. Databases of clinically relevant anatomical anomalies and normal anatomical variations could lead to a greater appreciation for the range of what is healthy and normal, opening up opportunities for discussions of the diversity of human anatomy. Photogrammetry methods such as WIDAR may serve as an additional tool for building an engaging and innovative learning experience for the good of all medical students.
